# Comparison between two different concentrations of ropivacaine in pericapsular nerve group block for patients undergoing total hip arthroplasty: A randomized clinical trial

**DOI:** 10.1371/journal.pone.0348565

**Published:** 2026-05-21

**Authors:** Chenxu Sun, Zhihua Sun, Dawei Liu, Dawei Chen, Ru Dong, Zhiyang Cai

**Affiliations:** 1 Department of Anesthesiology, Xiangya Changde Hospital, Changde, China; 2 Department of Anesthesiology, Xiangya Hospital, Central South University, Changsha, China; 3 Department of Anesthesiology, Changde City first Hospital of Traditionnal Chinese medicine, Changde, China; 4 Department of Anesthesiology, Hainan Modern Women And Children’s Hospital, Haikou, China; Asan Medical Center, University of Ulsan College of Medicine, KOREA, REPUBLIC OF

## Abstract

Effective pain control during spinal anesthesia positioning remains a clinical challenge in elderly patients undergoing total hip arthroplasty. The pericapsular nerve group block, a novel ultrasound-guided regional anesthesia technique, has shown promise for alleviating hip pain, yet the optimal concentration of local anesthetics for this approach remains unclear. In this randomized, observer-blinded clinical trial involving 102 patients, we compared the analgesic efficacy of two concentrations of ropivacaine (0.375% vs. 0.5%) administered via pericapsular nerve group block prior to spinal anesthesia. Patients receiving 0.5% ropivacaine reported significantly lower pain scores during positioning and demonstrated prolonged postoperative analgesia up to 48 hours, without a corresponding increase in adverse events. Both concentrations improved positioning quality, reduced opioid requirements, and enhanced recovery compared to controls who received standard intravenous analgesic supplementation (including preoperative midazolam and ketorolac, and postoperative sufentanil PCA), though the higher concentration yielded modest additional benefits. These findings suggest that a 0.5% ropivacaine pericapsular nerve group block may offer superior perioperative analgesia for hip arthroplasty without compromising safety, while highlighting the need to balance analgesic potency with potential motor effects. This study informs optimization of nerve block protocols to improve perioperative outcomes in a high-risk surgical population. This study informs optimization of nerve block protocols to improve perioperative outcomes in a high-risk surgical population. The trial was registered with the Chinese Clinical Trial Registry (registration number: ChiCTR2400089440).

## Introduction

The global rise in life expectancy has led to a projected increase in hip fractures, with estimates reaching 6.3 million cases annually within two decades [1]. These injuries carry substantial mortality risks in older adults, over 70% of whom are above 80 years of age. Patients in this age group often exhibit frailty, multiple comorbidities, and delayed recovery from anesthesia [2]. Total hip arthroplasty remains the definitive intervention to restore mobility and improve quality of life in this vulnerable population.

Spinal anesthesia is commonly used for total hip arthroplasty due to its minimal respiratory impact [3]. However, the sitting or lateral decubitus posture required for neuraxial block often induces substantial pain and distress during patient positioning [4]. The pericapsular nerve group block, a recently developed ultrasound-guided technique, has emerged as an effective strategy for mitigating acute hip pain in this setting [5,6].

The efficacy of regional nerve blocks depends critically on both the concentration and volume of the local anesthetic administered [7]. Despite increasing adoption of the pericapsular nerve group block in hip surgery, no clinical studies have directly compared the analgesic performance of different anesthetic concentrations in this technique.

In this randomized, observer-blinded trial, we evaluated whether the analgesic effect of the pericapsular nerve group block varies with the concentration of local anesthetic by comparing two concentrations of ropivacaine in patients undergoing total hip arthroplasty. The primary endpoint was pain during positioning for spinal anesthesia. Secondary outcomes included positioning quality, spinal anesthesia performance time, postoperative pain scores, opioid requirements, quadriceps strength, time to first analgesic request, recovery quality within 48 hours, and anesthesia-related complications.

## Materials and methods

### Study design and patients

This prospective, randomized, observer-blinded trial was conducted in the Department of Anesthesiology from January 20th, 2023 to October 10th, 2024, following the principles of the Declaration of Helsinki (2013 revision). The study was prospectively registered with the Chinese Clinical Trial Registry (registration number: ChiCTR2400089440) and received approval from the local ethics committee. Written informed consent was obtained from all participants, and patient confidentiality was strictly maintained throughout the study.

Eligible participants were adults aged 60–85 years with a body weight of 40–85 kg and an American Society of Anesthesiologists physical status of I to III, all scheduled for total hip arthroplasty. Exclusion criteria included refusal to participate, history of opioid abuse or psychiatric illness, significant pulmonary disease, coagulation disorders, allergy to local anesthetics, skin infection at the injection site, or preexisting peripheral neuropathy.

**Fig 1:**
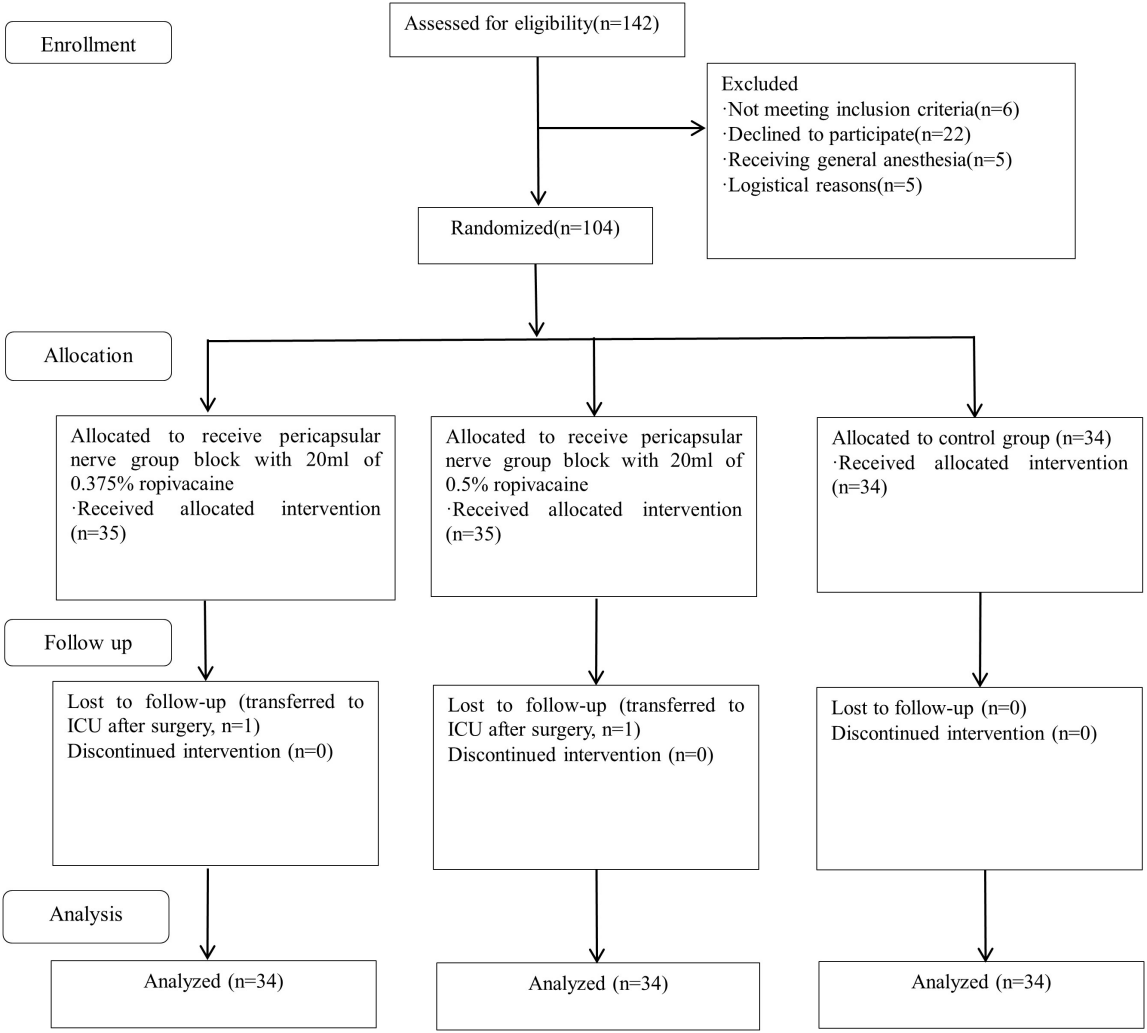
CONSORT flow diagram of patient enrollment, randomization, and analysis.

From January 2023 to October 2024, a total of 142 patients were screened for eligibility. Of these, 6 did not meet the inclusion criteria, 22 declined participation, 5 required general anesthesia, 5 were excluded for logistical reasons, and 2 were transferred to the intensive care unit postoperatively. In total, 104 patients were enrolled and randomized into the three study groups (Fig 1). Two patients (one from each intervention group) were transferred to intensive care postoperatively and excluded from analysis, leaving 102 patients for final analysis. Baseline characteristics were comparable across groups Table 1.

**Table 1:** Baseline demographic and perioperative characteristics of the three study groups.

Variables	PENG group (0.375%)	PENG group (0.5%)	Control group	P value
**n**	34	34	34	
**Age (years)**	73.4 ± 6.36	72.71 ± 7.03	73.74 ± 5.41	0.794
**Sex (F:M)**	14:20	10:24	12:22	0.597
**Height (cm)**	160.24 ± 6.04	157.82 ± 5.24	158.38 ± 5.79	0.194
**Weight (kg)**	61.97 ± 10.41	59.94 ± 9.77	60.82 ± 9.85	0.705
**Body mass index (kg/m²)**	23.76 ± 3.53	24.07 ± 3.82	24.2 ± 3.4	0.875
**ASA physical status (I:II:III)**	5:14:15	6:13:15	5:12:17	0.978
**Surgery duration (min)**	98.94 ± 10.53	100.24 ± 13.88	101.18 ± 13.28	0.766

PENG group (0.375%), 0.375% ropivacaine to perform PENG block; PENG group (0.5%), 0.5% ropivacaine to perform PENG block; Data are presented as mean ± standard deviation unless otherwise indicated. BMI, body mass index; ASA, American Society of Anesthesiologists.

Flow diagram illustrating patient enrollment, group allocation, follow-up, and analysis. Of the 142 patients assessed for eligibility, 104 were randomized into three groups: 0.375% ropivacaine (n = 35), 0.5% ropivacaine (n = 35), and control (n = 34). Two patients (one from each intervention group) were transferred to intensive care and excluded from final analysis.

### Randomization and blinding

After obtaining informed consent, patients were randomly allocated in a 1:1:1 ratio to one of three groups using a computer-generated simple randomization sequence. The sequence was generated by the principal investigators (C.S. and Z.C.). Allocation was concealed using sequentially numbered, opaque, sealed envelopes, which were prepared by an anesthesiologist not involved in subsequent study procedures. The envelopes contained the group assignments: 20 mL of 0.5% ropivacaine (HC group), 20 mL of 0.375% ropivacaine (LC group), or no regional block (control group). A control group (no regional block) was included for two reasons: first, to confirm the analgesic efficacy of PENG block even at the lower ropivacaine concentration; second, to help distinguish block-related motor effects from other perioperative factors, given the reported risk of quadriceps weakness. Patients in the HC and LC groups received a preoperative ultrasound-guided PENG block in a designated procedure room. The block was performed by a single anesthesiologist who had no further involvement in the study. To maintain observer blinding, the following personnel were kept unaware of group assignment: (1) the anesthesiologists performing intraoperative management; (2) the anesthesiologists and nurses providing postoperative care in the recovery unit; and (3) all research staff involved in outcome assessment (including pain scoring, quadriceps strength testing, and questionnaire administration). The block-performing anesthesiologist had no further role in the study.

### Study intervention

In both the block and operating rooms, standard monitoring, including noninvasive blood pressure, electrocardiography, and pulse oximetry, was initiated, and peripheral intravenous access was established. Pre-block pain levels were recorded using the numeric rating scale (NRS). All patients received intravenous midazolam (0.02 mg/kg) and ketorolac (30 mg) prior to the nerve block. Patients in the HC and LC groups then underwent ultrasound-guided PENG block administered by the same anesthesiologist.

**Fig 2:**
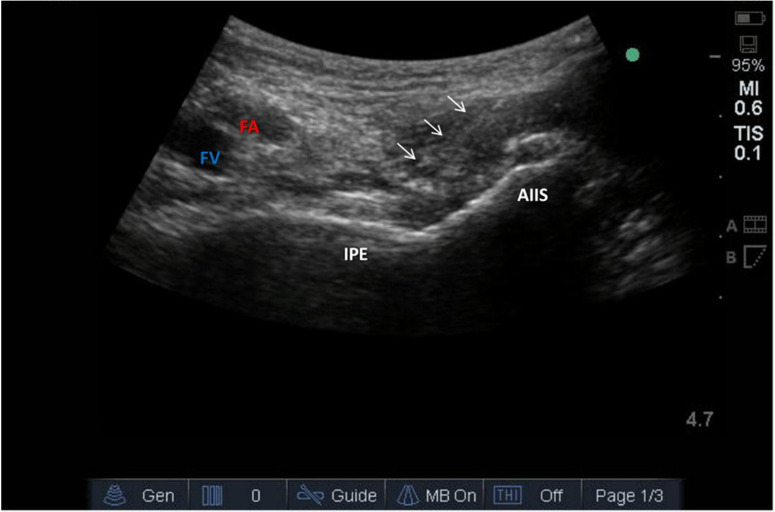
Ultrasound-guided anatomical landmarks for PENG block administration.

A low-frequency (2–5 MHz) curvilinear transducer was positioned transversely over the anterior inferior iliac spine to visualize target anatomy. Key landmarks included the pubic ramus, iliopubic eminence, iliopsoas muscle and tendon, and the femoral vessels (Fig 2). A 22G echogenic needle was inserted in-plane from lateral to medial, with its tip placed between the psoas tendon (anterior) and pubic ramus (posterior). Correct needle placement was confirmed by hydrodissection with 2 mL saline beneath the iliopsoas. After negative aspiration, 20 mL of 0.375% or 0.5% ropivacaine was administered. Patients were closely observed for signs of systemic toxicity or altered mental status. Patients in the control group received the identical preoperative and postoperative intravenous analgesic regimen as the two PENG block groups, but did not receive the ultrasound-guided block.

Ultrasound image demonstrating the anatomical landmarks used for pericapsular nerve group (PENG) block. AIIS, anterior inferior iliac spine; IPE, iliopectineal eminence; FA, femoral artery; FV, femoral vein. The white arrows indicate the in-plane trajectory of the needle tip during the procedure.

Following block assessment, patients were transferred to the operating room, positioned in the lateral decubitus position, and assessed for positioning-related pain. Spinal anesthesia was performed via epidural puncture at the L2/3 or L3/4 level, using 7.5–10 mg of bupivacaine. Postoperatively, all patients received intravenous patient-controlled analgesia (PCA) consisting of sufentanil (2 μg/kg) and ondansetron (8 mg) in 100 mL saline. The PCA device was programmed for a 2 mL/h background infusion, 1 mL on-demand bolus, 30-minute lockout, and 4 mL/h maximum dose, and was discontinued at 48 hours.

### Observation indicators

The primary outcome was the maximum numeric rating scale (NRS) pain score reported during patient positioning for spinal-epidural anesthesia. Prior to the procedure, patients were educated on the NRS assessment method and informed that they would be asked to rate their pain after assuming the lateral decubitus position. After the PENG block, patients were assisted by an anesthesiologist into the lateral decubitus position. Once the position was deemed suitable for spinal anesthesia, patients were instructed to maintain the posture, and the NRS pain score related to positioning was immediately assessed. Secondary outcomes included other perioperative NRS pain scores, quality of patient positioning, spinal anesthesia performance time, 48-hour opioid consumption, time to first analgesic request (PCA activation), quadriceps strength, quality of recovery, and anesthesia-related complications.

Positioning quality was graded as “bad,” “acceptable,” “satisfactory,” or “excellent,” corresponding to scores of 0–3. NRS pain scores were additionally recorded pre-block and at 6, 12, 24, and 48 hours postoperatively, during a standardized leg raise to 30 degrees. Prior to each assessment, patients were clearly instructed that 0 represented ‘no pain’ and 10 represented ‘the worst pain imaginable.’ They were then asked to self-report an integer from 0 to 10 corresponding to their current pain level. Quadriceps strength was evaluated 24 hours after surgery using the Oxford muscle strength scale: 0 (absent), 1–4 (reduced), or 5 (intact). Assessment was performed with the patient seated and the knee flexed. The patient was instructed to extend the leg against manual resistance provided by the assessor. Recovery quality was assessed using the Quality of Recovery-15 questionnaire at 24 hours postoperatively. Recovery quality was assessed at 24 hours postoperatively using the validated 15-item Quality of Recovery (QoR-15) questionnaire [8]. The QoR-15 evaluates five domains: physical independence, pain, physical comfort, psychological support, and emotional state, with a total score ranging from 0 (poor recovery) to 150 (excellent recovery).

All postoperative assessments (NRS pain scores, quadriceps strength, QoR-15 questionnaire, and documentation of adverse events) were conducted by a single trained research assistant who was blinded to group allocation and not involved in patient care. This was done to ensure objective outcome evaluation.

### Monitoring of adverse events

A predefined set of anesthesia- and block-related adverse events were systematically monitored and recorded. Local anesthetic systemic toxicity (LAST) was assessed intraoperatively by the attending anesthesiologist based on clinical signs (e.g., neurological symptoms such as perioral numbness, tinnitus, or seizures; cardiovascular symptoms such as arrhythmia or hypotension). Hemodynamic instability was defined as intraoperative hypotension (systolic blood pressure decrease >30% from baseline) or bradycardia (heart rate <60 beats per minute). Persistent neurological deficit was defined as any new sensory, motor, or autonomic dysfunction persisting for more than 3 months postoperatively. Neurological status was assessed before discharge, and any abnormality triggered follow-up. Postoperative nausea and vomiting (PONV) and dizziness were recorded during standardized patient interviews on postoperative days 1, 2, 3, and at discharge. Quadriceps motor weakness was assessed as part of the scheduled 24-hour muscle strength evaluation.

### Sample size and statistical analysis

As no previous studies had directly compared different concentrations of ropivacaine in the PENG block, we conducted a pilot study involving 12 patients. The maximum positioning-related pain scores were 4.00 ± 1.80 in the 0.375% group and 2.00 ± 1.90 in the 0.5% group. Based on the pilot comparison between the two PENG groups (0.375% vs. 0.5%), with a significance level of 5% and statistical power of 95%, the minimum required sample size was calculated to be 28 patients per PENG group. To accommodate an anticipated 20% dropout rate, we enrolled 34 patients in each group.

Statistical analyses were performed using SPSS version 26.0 (IBM Corp., Armonk, NY, USA). Data normality was assessed using the Shapiro-Wilk test. Continuous variables are described according to their distribution: normally distributed data as mean ± standard deviation, and non-normally distributed data as median (interquartile range). This reporting scheme is applied consistently throughout the manuscript. One-way analysis of variance was used for group comparisons of normally distributed data, and the Kruskal-Wallis test for non-normally distributed data. Categorical variables were analyzed using Pearson’s χ² test. Kaplan-Meier analysis was employed to assess differences in time to first analgesic request. Given that postoperative NRS pain scores were assessed at multiple time points, the repeated-measures nature of this data was accounted for in the statistical modeling. A two-sided p-value < 0.05 was considered statistically significant.

## Results

**Fig 3:**
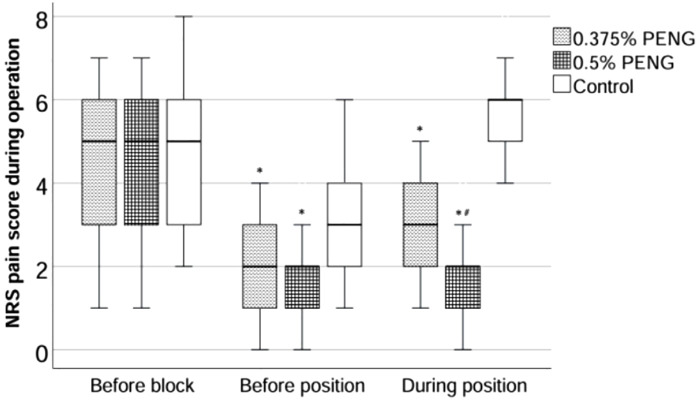
NRS pain scores before and after PENG block and during positioning for spinal anesthesia.

Pre-block NRS pain scores did not differ significantly among groups. However, both the high-concentration (HC) and low-concentration (LC) PENG groups exhibited significantly lower pain scores before and during positioning for spinal anesthesia compared to the control group (Fig 3). Moreover, the HC group reported lower positioning pain scores than the LC group (p = 0.03), although no significant difference was observed between the groups in post-block, pre-positioning pain (p = 0.761). The quality of patient positioning and the time required to perform spinal anesthesia were significantly improved in both PENG groups versus control, with no differences between the HC and LC groups.

Box plots of numeric rating scale (NRS) pain scores in the three study groups—before the PENG block, after the block but prior to positioning, and during positioning for spinal anesthesia. *p < 0.05 versus control group; # *p* < 0.05 versus 0.375% PENG group.

**Fig 4:**
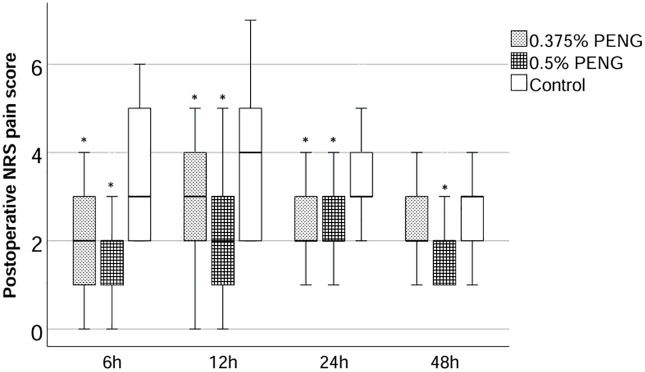
Postoperative NRS pain scores at 6, 12, 24, and 48 hours across the three study groups.

At 6, 12, and 24 hours postoperatively, pain scores were significantly lower in both PENG groups relative to the control group, with no differences between the HC and LC groups (Fig 4). At 48 hours, the HC group continued to demonstrate reduced pain compared to the control (p = 0.01), whereas the LC group showed no significant difference from either the control (p = 0.384) or HC group (p = 0.09). To appropriately analyze the repeated postoperative NRS pain scores, a Generalized Estimating Equations (GEE) model was employed. This analysis indicated that the trajectory of pain scores was significantly influenced by both time and treatment group, with a significant time-by-group interaction (all P < 0.01). In line with this model, the specific between-group comparisons at each time point were evaluated. At 6, 12, and 24 hours postoperatively, pain scores were significantly lower in both PENG groups relative to the control group, with no differences between the HC and LC groups (Fig 4). At 48 hours, the HC group continued to demonstrate reduced pain compared to the control (p < 0.01), whereas the LC group showed no significant difference from either the control (p = 0.343) or HC group (p = 0.089).

Box plots of postoperative numeric rating scale (NRS) pain scores at 6, 12, 24, and 48 hours in the 0.375% PENG, 0.5% PENG, and control groups. *p < 0.05 vs. control group; # *p* < 0.05 vs. 0.375% PENG group.

The following secondary outcomes were significantly improved in both PENG groups compared to control: quality of positioning, spinal anesthesia performance time, 48-hour cumulative opioid use, time to first analgesic request, and Quality of Recovery-15 scores (Table 2).

**Table 2:** Perioperative outcomes and postoperative recovery metrics across the three study groups.

	PENG group (0.375%)	PENG group (0.5%)	Control group	P value
**Positioning quality (score)**	2(2-3)*	3(2-3)*	2(1-2)	—
**Spinal anesthesia performance time (min)**	3.76 ± 0.99*	3.82 ± 0.93*	4.64 ± 1.35	—
**Sufentanil consumption (µg)**	110.35 ± 17.48*	108.53 ± 13.8*	119.41 ± 16.6	—
**Time to first analgesic request (h)**	8.18 ± 2.61*	7.85 ± 2.18*	6.18 ± 2.53	—
**Quality of recovery (QoR-15 score)**	115.32 ± 11.76*	113.68 ± 11.07*	99.29 ± 12.42	—
**Quadriceps strength (intact/reduced/absent)**	29/5/0	27/6/1	33/1/0	0.19
**LAST, n**	0	0	0	—
**Hypotension**	9	10	8	0.860
**Bradycardia**	3	4	4	0.903
**PONV, n (%)**	6(17.6%)	5(14.7%)	8(23.5%)	0.646
**Dizziness, n (%)**	2(6%)	2(6%)	3(9%)	0.858

**Fig 5:**
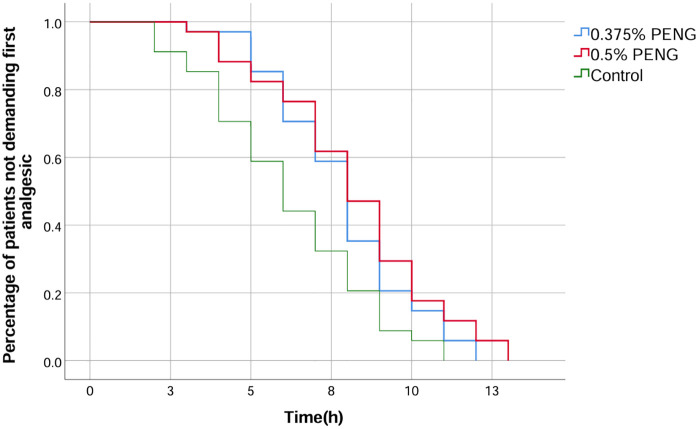
Kaplan–Meier curves of time to first analgesic request after surgery in each study group.

Data are presented as mean ± SD, median (IQR), or count (%), as appropriate. LAST, local anesthetic systemic toxicity; PONV, postoperative nausea and vomiting; SD, standard deviation; IQR, interquartile range. p < 0.05 vs. control group;

However, no significant differences were observed between the HC and LC groups for any of these measures. Kaplan-Meier analysis confirmed a significant difference in the time to first analgesic request across the three groups (Fig 5). Quadriceps strength at 24 hours and the incidence of anesthesia-related adverse events were similar across groups Table 2.

Kaplan–Meier survival curves illustrating the time to first analgesic request following surgery in the 0.375% PENG, 0.5% PENG, and control groups. The y-axis represents the proportion of patients not requiring analgesia at each time point.

## Discussion

This randomized trial assessed the efficacy of two concentrations of ropivacaine (0.375% vs. 0.5%) at a fixed volume (20 mL) for pericapsular nerve group (PENG) block in patients undergoing total hip arthroplasty. The 0.5% concentration provided superior analgesia during spinal anesthesia positioning and sustained pain relief at 48 hours compared with the 0.375% group. The potential influence of surgical duration on postoperative outcomes was also considered. Prolonged operative time is generally associated with greater surgical trauma, inflammatory response, and physiological stress, which could independently worsen pain and delay recovery. In this study, however, operative time was well-balanced across all three groups, with no statistically significant difference (Table 1). This balance suggests that surgical duration is unlikely to be a major confounding factor affecting the comparison of postoperative pain scores and recovery metrics between the groups.

Neuraxial techniques are considered optimal for hip fracture surgeries; however, patient positioning for spinal anesthesia often exacerbates hip pain and elicits a stress response. Such discomfort can compromise anesthetic success and destabilize hemodynamics, critically impacting older adult [9]. While various strategies exist to reduce positioning pain, regional nerve blocks have demonstrated greater efficacy than systemic analgesia [10]. As a relatively recent musculofascial technique, the PENG block is increasingly favored for pre-spinal anesthesia analgesia. Previous studies suggest that PENG offers superior pain control and better preserves quadriceps strength than femoral or fascia iliaca blocks [11–13]. However, the concentration of local anesthetics used across these studies has varied, including 0.375%, 0.5%, and 0.75% ropivacaine [13–15] as well as 0.125% and 0.25% bupivacaine [4,12].

Our results suggest that 0.5% ropivacaine provides modest but clinically relevant advantages over 0.375% in relieving positioning-related pain. The clinical relevance of this statistically significant difference merits consideration. The observed between-group difference in NRS scores during positioning ranged from 1 to 2 points across our pilot and main studies. In the context of acute pain, the established minimal clinically important difference for patient-reported pain on the NRS is approximately 1.3 points [16]. Thus, the difference observed in our trial meets or approaches this threshold. It is noteworthy that the larger difference (2.0 points) observed in our pilot study was not fully replicated, suggesting that the incremental analgesic benefit of the higher concentration may be modest and subject to individual variability. In clinical practice, this finite additional benefit must be carefully weighed against the potential risk of quadriceps motor impairment associated with the 0.5% concentration, particularly for patients in whom early postoperative mobilization is a priority. The 10-minute interval between block administration and positioning, though commonly used [4,15,17] may be insufficient to capture maximal analgesic effect. Other studies have adopted longer waiting times [11,18], and the optimal onset interval for the PENG block remains undetermined. The greater analgesic effect seen with the higher concentration may reflect faster onset or stronger nerve blockade, which warrants further pharmacodynamic investigation. Our results suggest that the analgesic efficacy of the PENG block is correlated with the concentration of ropivacaine within the range tested. However, a formal dose-response relationship cannot be established from comparing only two concentrations at a fixed volume, as the total drug dose was not an independent variable.

Although the 0.5% concentration provided statistically superior analgesia during positioning, the clinical relevance of this difference warrants consideration. The between-group difference in NRS scores (1–2 points) approaches the minimal clinically important difference (MCID) of approximately 1.3 points established for acute pain. Notably, the larger difference observed in our pilot study was not fully replicated, suggesting that the incremental benefit of the higher concentration may be modest and variable among individuals. And our results suggest that no significant differences were observed between the HC and LC groups for any of other outcomes. This pattern suggests that a concentration of 0.375% ropivacaine may already provide a satisfactory analgesic effect for improving perioperative pain in these patients. Therefore, when choosing the concentration for PENG block, clinicians should weigh this potential analgesic advantage against the risk of motor impairment, particularly in patients requiring early ambulation. To evaluate the duration of postoperative analgesia, we included a control group. Analgesia in the 0.5% group persisted up to 48 hours, while the 0.375% group achieved comparable relief only up to 24 hours. These findings are consistent with prior trials using similar concentrations of ropivacaine or bupivacaine [18,19].

Although direct evidence for concentration–response relationships in PENG block is limited, studies of other peripheral nerve blocks support a correlation between higher anesthetic concentration and longer analgesia duration [20,21]. In our study, both PENG groups demonstrated reduced opioid consumption and delayed time to first analgesic request compared with controls, aligning with previous reports [22–24]. However, these metrics did not differ significantly between the two concentrations, suggesting diminishing returns beyond a certain dose threshold.

While increasing anesthetic volume has been explored to enhance PENG efficacy, results have been mixed. Sharma et al. found that 20 mL of 0.25% bupivacaine improved positioning quality compared to 15 mL [25], but other studies reported no added benefit with 30 mL versus 20 mL [26]. Excess volume may lead to unintended spread to the sacral plexus or adjacent structures [27, 28]. Based on this, we selected 20 mL for both concentrations in our study. Both PENG groups showed improved positioning and reduced spinal anesthesia duration compared with controls; however, increasing ropivacaine concentration did not further enhance these procedural metrics.

High concentrations of local anesthetics raise concerns about neurotoxicity and motor blockade, which may delay mobilization [20,29]. Although quadriceps strength at 24 hours showed no significant intergroup difference, reduced strength occurred more frequently in both PENG groups, particularly in the 0.5% group, where one patient developed transient complete motor block. All patients recovered full strength by 48 hours. Previous studies have also reported transient quadriceps weakness following PENG block, possibly due to spread to the femoral or fascia iliaca compartments [26]. These observations underscore the need for caution in patients requiring early ambulation postoperatively. The potential for quadriceps weakness holds direct clinical relevance. Within ERAS protocols that emphasize early ambulation, such motor impairment can delay postoperative mobilization. Our data show that a notable number of patients in the PENG groups exhibited reduced strength at 24 hours. Therefore, when planning analgesia, the benefit of the block must be balanced against the risk of transiently affecting motor function, particularly for patients in whom immediate ambulation is critical. Of note, not all relevant nerve block studies support the use of higher local anesthetic concentrations. For instance, one study in total knee arthroplasty reported that a lower concentration of bupivacaine used in a triple-nerve block provided more satisfactory analgesia than a higher concentration [30].

This study has several limitations. First, we did not perform serial NRS assessments following the block, and the fixed 10-minute interval before positioning may have been suboptimal. Second, hospital length of stay, a meaningful recovery indicator, was not assessed. Third, although powered for the primary endpoint, the sample size may have been insufficient to detect differences in secondary outcomes such as opioid use or time to analgesia. Finally, given the evolving adoption of the PENG block, longer-term follow-up is warranted to monitor for delayed or rare complications. Additionally, we did not collect objective physiological data, such as changes in blood pressure or heart rate during positioning, which could have provided further evidence of the pain response. Future studies may consider incorporating such measures to complement patient-reported outcomes. Furthermore, quadriceps strength was assessed only at 24 hours postoperatively. While this time point is used in several similar studies, it may not capture earlier motor block that could impede immediate postoperative mobilization within ERAS pathways. Future studies should consider evaluating motor function at earlier intervals (e.g., 6–12 hours) to better assess its impact on functional recovery. Third, it is important to note that all patients, including those in the control group, received a standardized background of systemic analgesia (preoperative midazolam and ketorolac, and postoperative sufentanil PCA). While this approach was ethically mandated to ensure patient comfort and safety, and the regimen was consistent across groups to allow for fair comparison, the systemic effects of these medications constitute a potential confounder. The contribution of these adjuncts to the overall pain relief observed, particularly in the control group, means that the measured treatment effect should be interpreted as the incremental benefit of the PENG block over and above this standard analgesic baseline, rather than an absolute effect compared to a pure ‘no analgesia’ state. Future studies aiming to isolate the pure effect of a peripheral nerve block might consider different methodological approaches, while balancing ethical constraints. Fourth, all patients received a standardized background of systemic analgesia, including preoperative ketorolac and postoperative sufentanil patient-controlled analgesia (PCA). This regimen was ethically mandated to ensure patient comfort and safety and represents standard perioperative care. However, it potentially masks the full magnitude of the treatment effect attributable to the PENG block alone and serves as a confounding factor. The analgesic contribution of these systemic medications, particularly the fixed, non-weight-adjusted dose of ketorolac, may have reduced the observable differences in pain scores between the intervention and control groups. Consequently, our results should be interpreted as demonstrating the additional or incremental benefit of the PENG block when integrated into a contemporary multimodal analgesic regimen. Finally, this study has inherent limitations regarding generalizability. As a single-center trial with a standardized perioperative protocol, our findings are derived from a specific patient population and a consistent care environment. The enrolled patients were within a defined age and physical status range (ASA I-III). Consequently, the results may not be directly applicable to all patients undergoing hip arthroplasty, particularly those with characteristics outside our study criteria or in centers with different perioperative practices. The external validity of our conclusions warrants confirmation through future multicenter studies.

### Clarification on pain assessment type

It is important to note that the pain scores reported in this study specifically reflect activity-related pain (during positioning and active leg raise), not pain at rest. This focus was chosen because pain upon movement is a primary barrier to functional recovery and early mobilization after hip arthroplasty, making it a highly relevant clinical endpoint. While the assessment of rest pain is valuable and employed in other studies of regional anesthesia for hip surgery [31], we prioritized the evaluation of dynamic pain to better align with functional recovery goals. However, we acknowledge that the inclusion of standardized rest pain assessments would have provided a more complete analgesic profile and enhanced comparability with the broader literature. Future studies may consider evaluating both dimensions to fully capture the perioperative analgesic effect of PENG blocks.

## Conclusions

In patients undergoing total hip arthroplasty, the pericapsular nerve group block using 0.5% ropivacaine provided more effective analgesia for positioning during spinal anesthesia and offered extended postoperative pain relief compared with 0.375% ropivacaine. However, potential quadriceps motor impairment associated with this higher concentration should be carefully considered in patients requiring early mobilization.

## Supporting information

S1 Table CONSORT 2025 editable checklist. (DOCX)

S2 Table PLOSOne Human Subjects Research Checklist. (DOCX)

S3 Table Informed consent form and ethical approval document. (DOCX)

S4 Table Trial study protocol. (DOCX)

## Abbreviations

PENG: Pericapsular nerve group
NRS: Numeric rating scale
AIIS: Anterior inferior iliac spine
IPE: Iliopubic eminence
ASA: American Society of Anesthesiologists
PCA: Patient-controlled analgesia
BMI: Body mass index
SD: Standard deviations
IQR: Interquartile range
