# Supplementary material for: Comparison between two different concentrations of ropivacaine in pericapsular nerve group block for patients undergoing total hip arthroplasty: A randomized clinical trial
Source: PLoS One. 2026 May 21;21(5):e0348565. doi: 10.1371/journal.pone.0348565 (PMC13193387; doi:10.1371/journal.pone.0348565)

Letter of Consent and Knowledge

You are invited to participate in a research study organized by the Department of Anesthesiology at Xiangya Changde Hospital. This study examines the perioperative effects of ultrasound-guided hip joint local anesthesia combined with spinal anesthesia in patients undergoing hip replacement surgery. The procedure involves enhancing conventional knee injection methods with color Doppler ultrasound guidance technology.

As your surgical plan and physical condition meet the eligibility criteria, we invite you to join this trial. This informed consent document provides essential information to help you decide whether to participate. Please read the following details carefully:

This research aims to investigate the effects of ultrasound-guided hip joint anesthesia combined with spinal-epidural anesthesia on multiple clinical parameters in hip replacement patients, including anesthesia induction pain scores, local anesthetic dosage for spinal anesthesia, procedural duration of spinal anesthesia, sensory block levels, perioperative cardiopulmonary changes, postoperative urinary function recovery, and immediate postoperative cognitive function.

1. Research process and methods: Ultrasound or C-arm guidance technology is added to conventional combined spinal-epidural anesthesia to evaluate the beneficial effects on this therapeutic approach.

Potential benefits for the researchers: (1). Effectively reducing the pain score during the anesthesia induction period for patients undergoing hip replacement surgery. (2) Reducing the dosage of local anesthetics used in spinal anesthesia, the duration of spinal anesthesia procedures, and maintaining a low level of anesthesia. (3). Minimizing fluctuations in the circulatory and respiratory systems during the perioperative period. (4). Promoting the recovery of urinary system function after surgery. (5). Enhancing early postoperative cognitive function improvement.

2. For the treatment described in the study, our department does not charge any procedural fees.

3. Major risks: Allergy to local anesthetics, local anesthetic toxicity, bleeding, and infection, etc. We will address these risks by having professional physicians administer treatment and using ultrasound-guided puncture to minimize damage. Even if they occur, our department will promptly take targeted measures. These risks are relatively minor compared to the benefits.

Participants have the right to decide whether to join or decline this trial, and they may withdraw at any time. Withdrawal for any reason will not affect the doctor-patient relationship, and participants will receive prompt medical treatment.

The privacy rights of participants will be protected.

The right to be informed of participants will be protected.

If participants suffer harm as a result of the trial, the sponsor will provide free treatment and offer financial compensation.

Subject's signature：
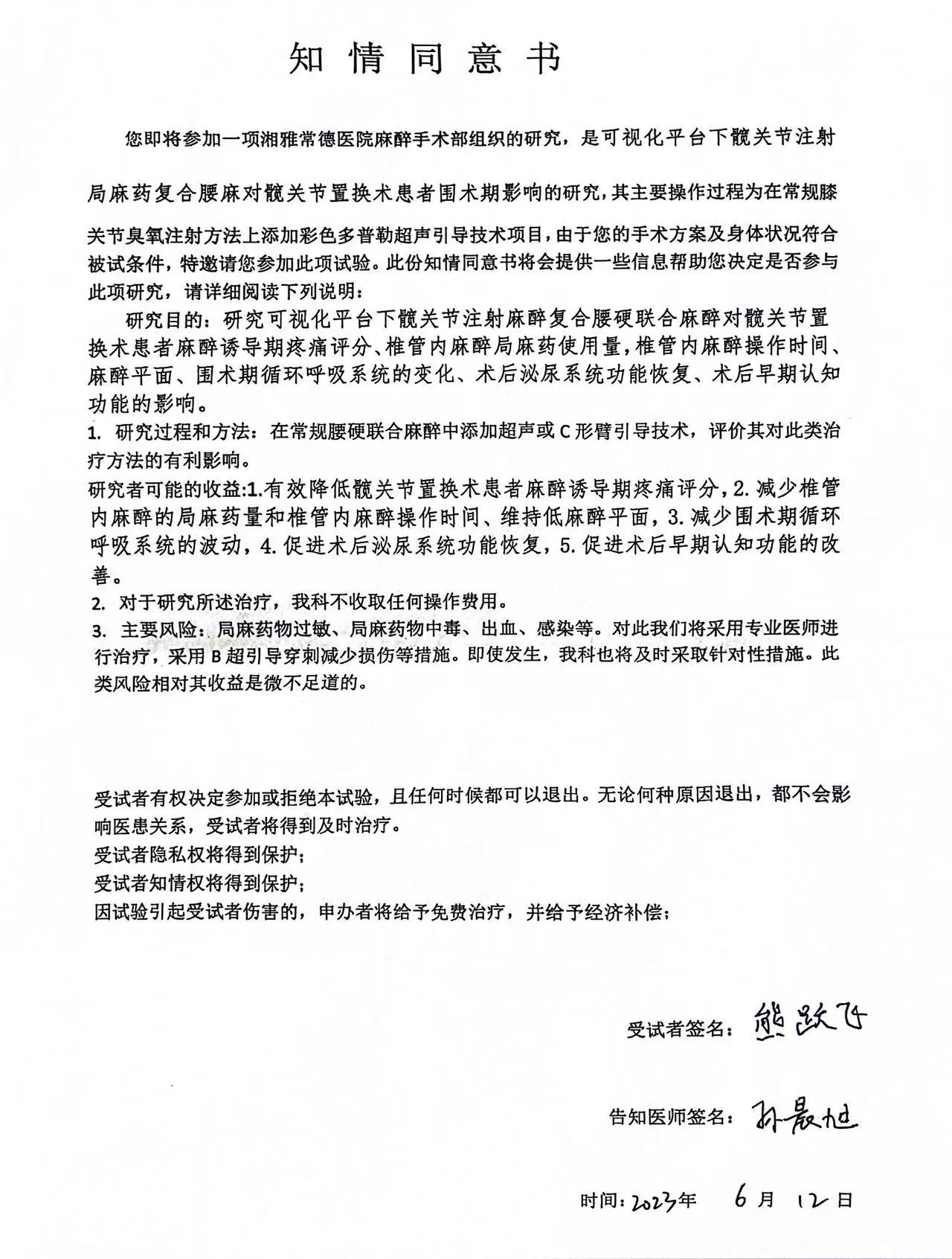


Inform the physician to sign：
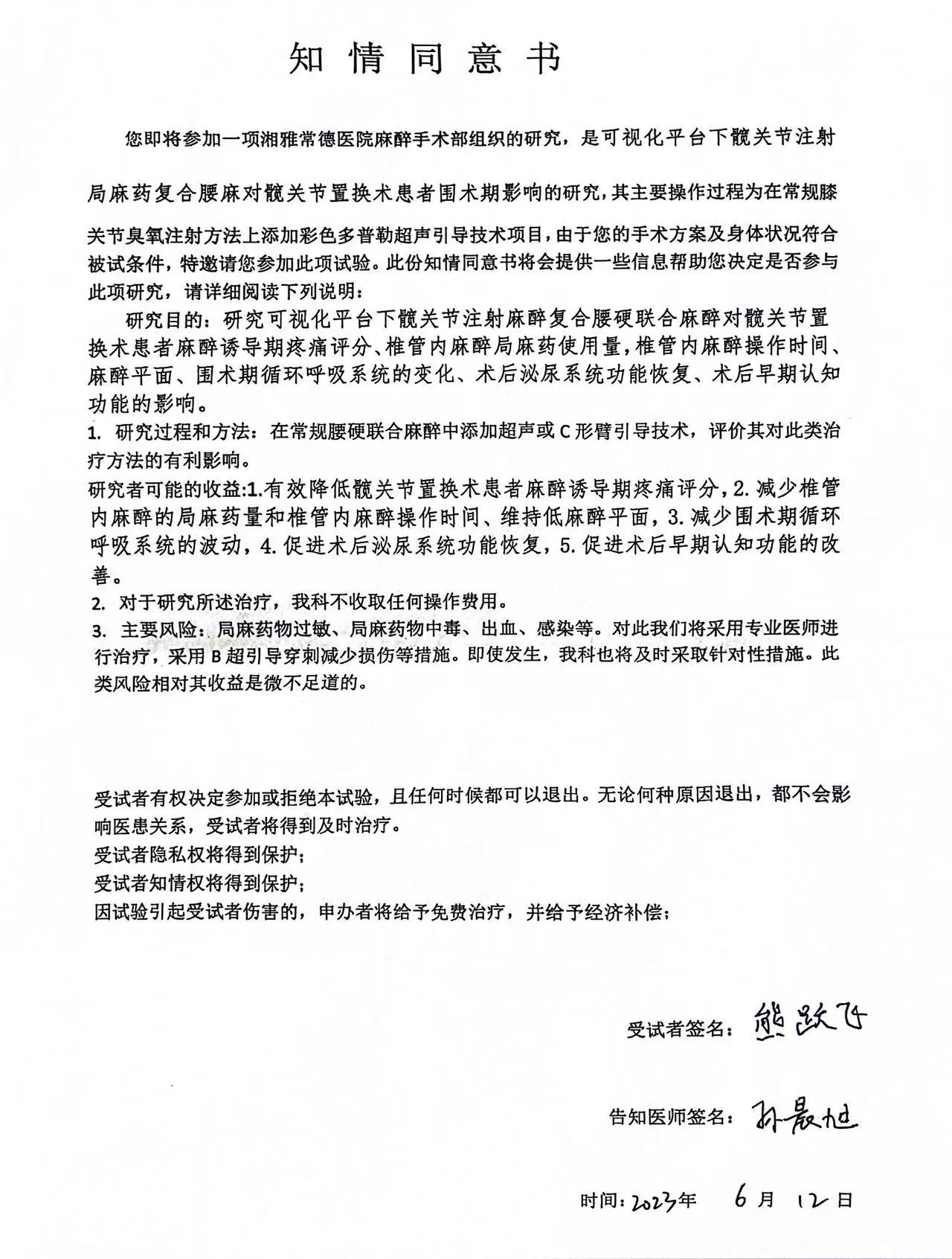


2023 / 06 / 12

Scientific Research Medical Ethics Review Form of the Medical Ethics Committee of Xiangya Changde Hospital

| Project Name | The impact of injecting local anesthetic combined with spinal anesthesia in the visualization platform on the perioperative period of patients undergoing hip replacement surgery | | | |
| --- | --- | --- | --- | --- |
| Assume the role of a research institution and a professional position | | Xiangya Changde Hospital  Anesthesiology Department | | |
| Take responsibility | | Responsible √ /Participate in the meeting | Principal investigator | Zhiyang Cai,  Zhihua Sun,  Zhenghua He, |
| Research classification | | 5 | Research start and end dates | January 2023 - December 2025 |
| Research type | | √ Clinical Research / Basic research | | |
| Proposed project type | | Research Project of the Health Commission of Hunan Province | | |
| Review Opinion | | √ Agree to declare / Disagree to declare | | |
| This study focuses on the main aspects of research ethics:  The study investigated the effects of hip joint injection of long-acting local anesthetic combined with spinal-epidural combined anesthesia on the pain score during the anesthesia induction period, the amount of local anesthetic used for spinal anesthesia, the operation time of spinal anesthesia, the anesthesia plane, the changes in the circulatory and respiratory systems during the perioperative period, the recovery of postoperative urinary system function, and the early cognitive function of patients undergoing hip replacement surgery under this visualization platform.  Ethical review opinion:  After review by our hospital's ethics committee, the experimental design and plan of this study fully consider the principles of safety and fairness. The research content will not cause harm or risks to the research subjects. The research subjects will be based on the principle of voluntary participation, and their right to know is guaranteed. The rights and privacy of the research subjects will be maximally protected. There is no conflict of interest between the research content and the research results. | | | | |
| Note: Research classification: 1 = Utilization of pathological tissues; 2 = Human experiments; 3 = Human tissue or cell experiments; 4 = Embryo cloning research; 5 = Others | | | | |


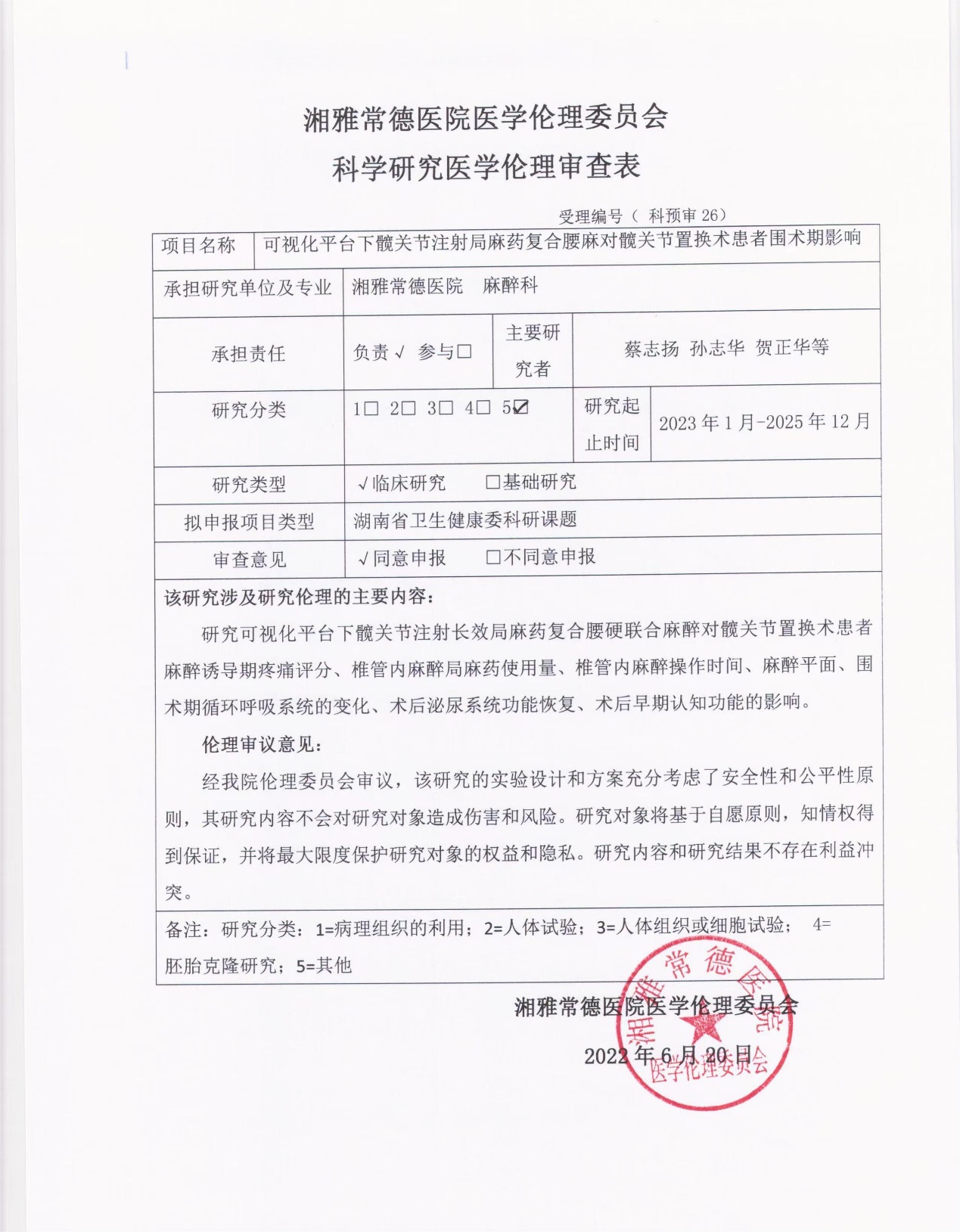
Xiangya Changde Hospital Medical Ethics Committee


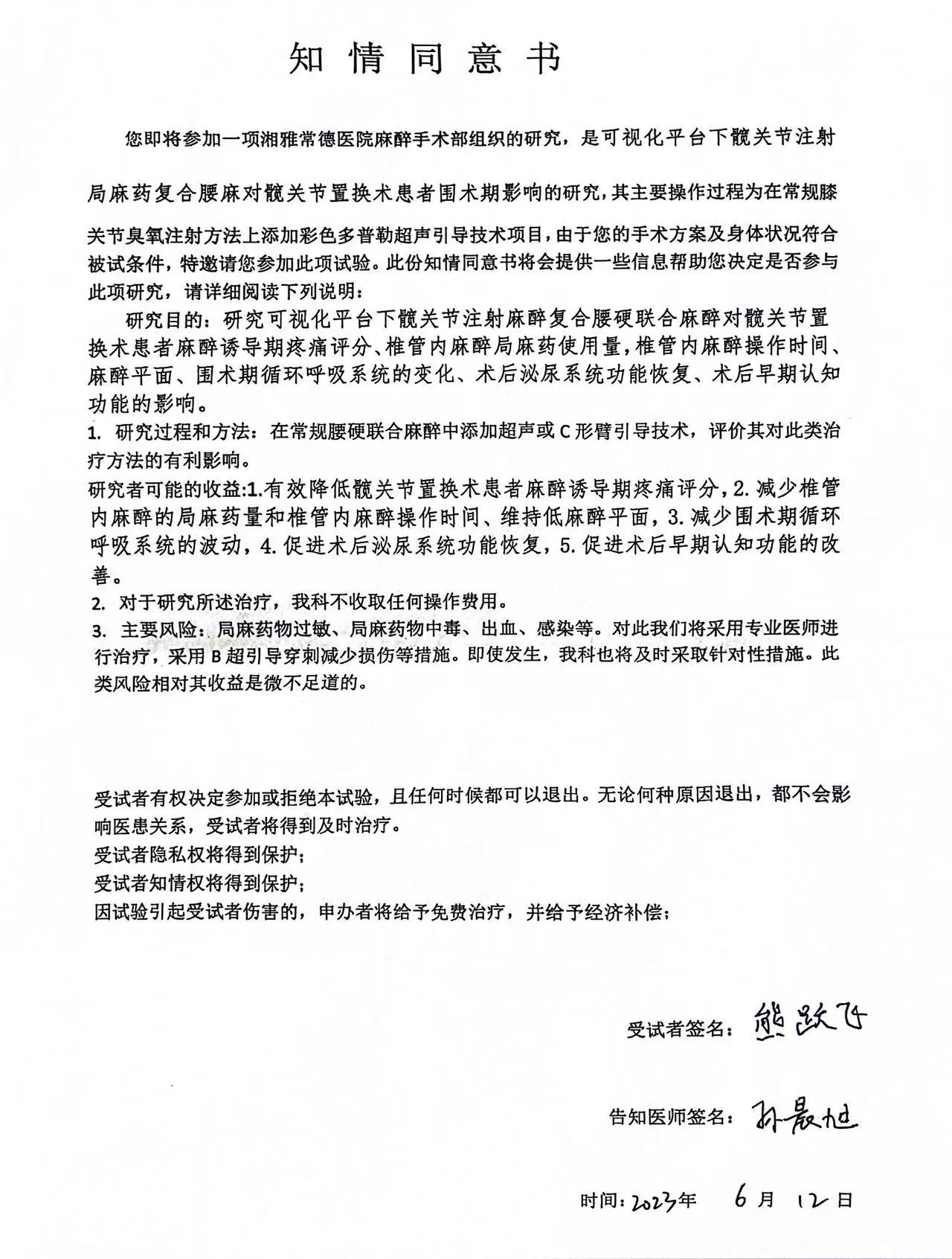


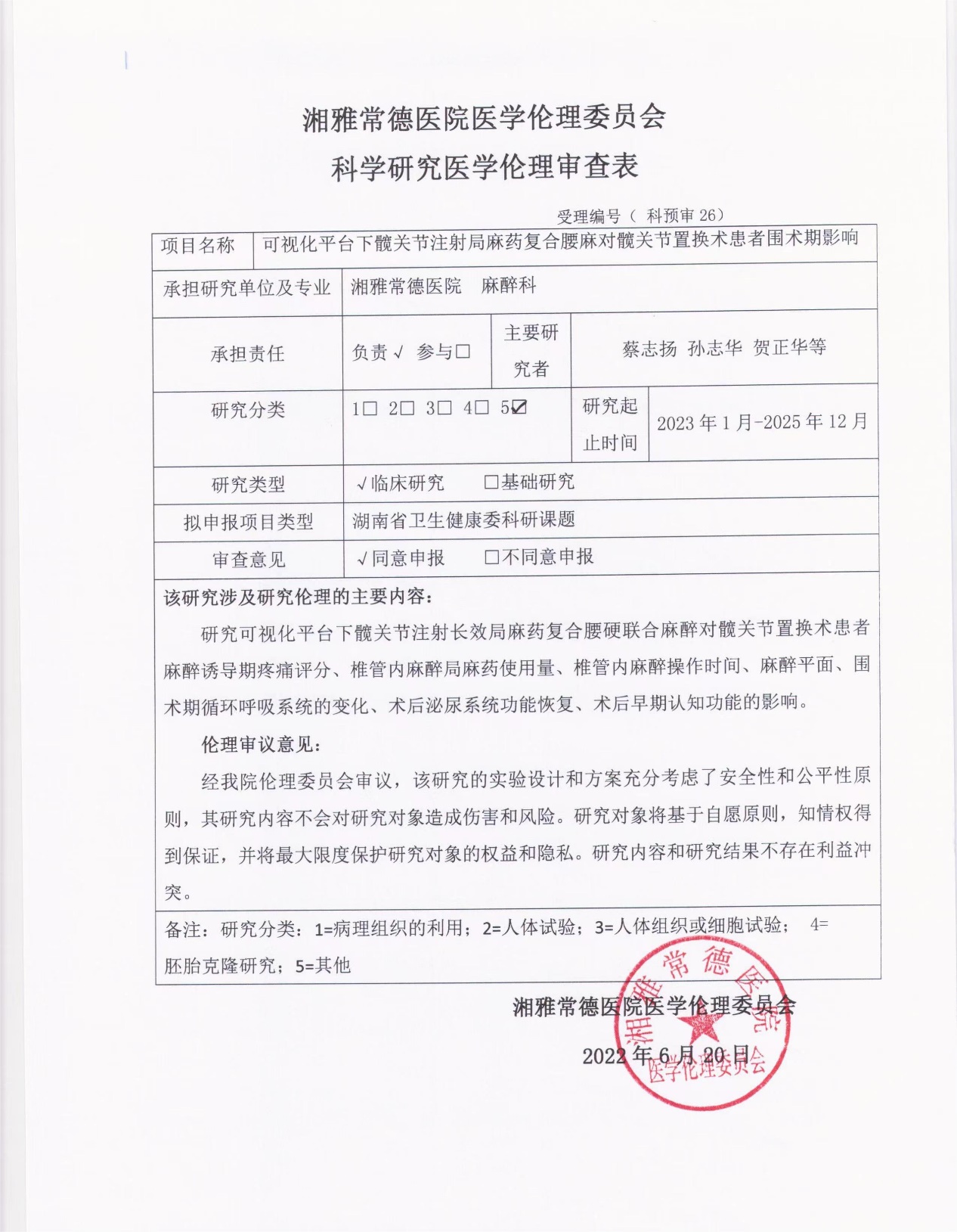


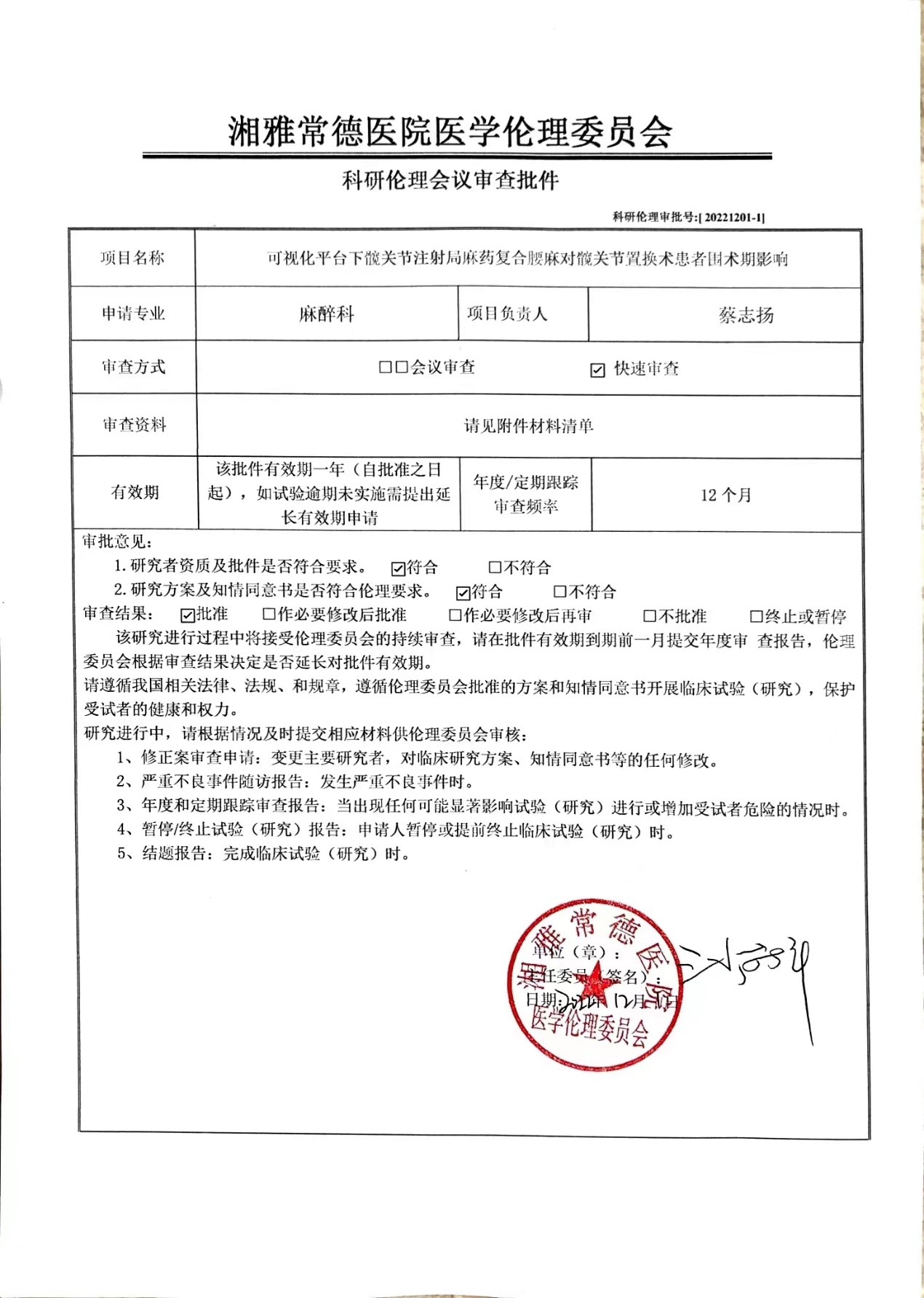

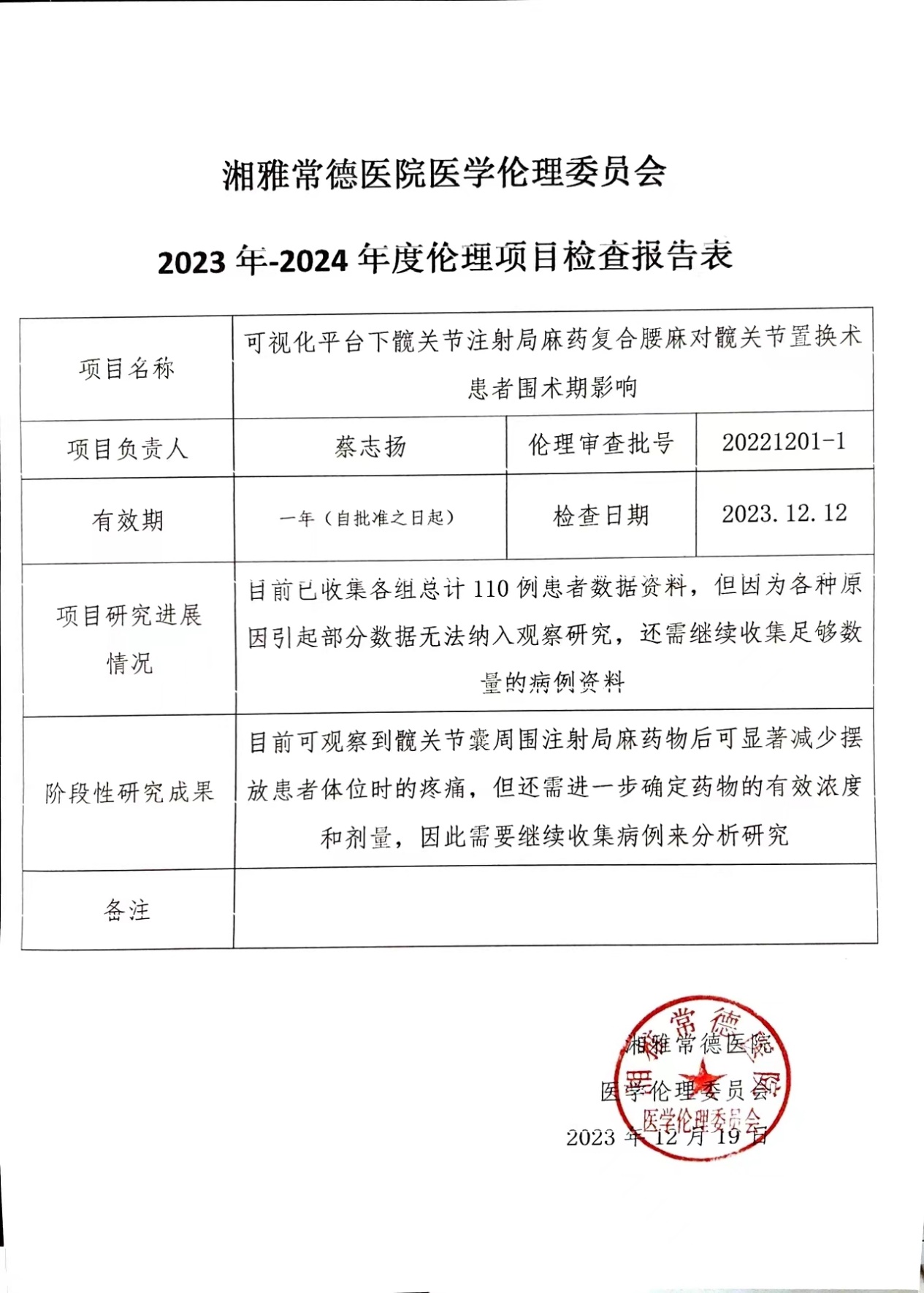

Supplement: S3 Table — (DOCX) [file pone.0348565.s003.docx]
