# Supplementary material for: Comparison between two different concentrations of ropivacaine in pericapsular nerve group block for patients undergoing total hip arthroplasty: A randomized clinical trial
Source: PLoS One. 2026 May 21;21(5):e0348565. doi: 10.1371/journal.pone.0348565 (PMC13193387; doi:10.1371/journal.pone.0348565)
Supplement: S4 Table — (DOCX) [file pone.0348565.s004.docx]

**Experimental Background**

Femoral neck fractures are prevalent among elderly patients, and hip replacement surgery is a common surgical treatment option. To ensure a successful surgery, various clinical anesthesia methods are available, including general anesthesia, combined spinal - epidural anesthesia, and nerve block [1].

Combined spinal - epidural anesthesia has several advantages, such as good analgesia, rapid drug onset, complete block, muscle relaxation, and the ability to maintain spontaneous breathing. It also reduces perioperative anesthesia-related complications and patient mortality compared to general anesthesia [2]. However, this method has certain challenges, especially in elderly patients. The positioning required for combined spinal-epidural anesthesia is more demanding due to the use of a thinner needle. Elderly patients often experience intense pain from fracture end friction during positioning. Moreover, their fear of the procedure can further complicate the anesthesia operation, leading to a prolonged operation time, a lower puncture success rate, and potentially a change in the anesthesia method.

Elderly patients frequently suffer from cardiovascular and cerebrovascular diseases, as well as neurological and respiratory system disorders. Intense pain before the effective entry of local anesthetics into the spinal canal can cause significant hemodynamic fluctuations, increasing the risk of perioperative cardiovascular and cerebrovascular adverse events [3].

In the past, intravenous analgesics, mainly potent opioids like sufentanil and fentanyl, were used before spinal anesthesia. However, elderly patients show individual differences in their response to opioids, resulting in either incomplete analgesia or side effects such as respiratory depression and excessive sedation.

In recent years, ultrasound-guided fascia iliaca compartment block (FICB) and pericapsular nerve group (PENG) block have been employed for treatment [4-5]. These nerve blocks can effectively reduce pain scores during different stages of the procedure, improve the quality of positioning, shorten the spinal anesthesia operation time, reduce the amount of spinal anesthetic drugs, and have positive impacts on postoperative urinary system function and the prevention of postoperative cognitive dysfunction [6].

Despite the potential benefits of PENG block, there is a lack of research in the literature regarding the appropriate concentration and volume of anesthetic drugs. This study aims to perform ultrasound-guided PENG block in hip replacement surgery using different concentrations of local anesthetic drugs before spinal anesthesia, and to compare the anesthetic effects among different groups.

[1] Yang Liu, Mang Su, Wei Li, et al. Comparison of general anesthesia with endotracheal intubation, combined spinal-epidural anesthesia, and general anesthesia with laryngeal mask airway and nerve block for intertrochanteric fracture surgeries in elderly patients: a retrospective cohort study. BMC Anesthesiol. 2019; 19(30). DOI: 10.1186 /s12871-019-0908-2.

[2] Pu Ma, Haibo Zeng. Effects of epidural anesthesia combined with dexmedetomidine on blood pressure, sedation, analgesia and serum β-endorphin levels in patients with hip fractures.Am J Transl Res. 2021; 13(6): 6457–6467.

[3] Afsaneh Norouzi, Fozhan Behrouzibakhsh, Alireza Kamali, et al. Short-term complications of anesthetic technique used in hip fracture surgery in elderly people. Eur J Transl Myol. 2018 Jul 10; 28(3): 7355.  DOI: 10.4081/ejtm.2018.7355

[4] Ya-Li Wang, Yun-Qing Liu, Hua Ni, et al. Ultrasound-guided, direct suprainguinal injection for fascia iliaca block for total hip arthroplasty: A retrospective study. World J Clin Cases. 2021; 9(15): 3567–3575.DOI: 10.12998/wjcc.v9.i15.3567

[5] C. Shelton, S. White. Anaesthesia for hip fracture repairBJA Educ. 2020; 20(5): 142–149. 23. DOI: 10.1016/j.bjae.2020.02.003

[6] André Strahl, Murteza Ali Kazim, Nils Kattwinkel, et al. Mid-term improvement of cognitive performance after total hip arthroplasty in patients with osteoarthritis of the hip: a prospective cohort study. Bone Joint J. 2022; 104-B(3): 331–340. DOI: 10.1302/0301-620X.104B3.BJJ-2020-2021.R2

**Method**

The experiment will enroll patients with femoral neck fractures who are scheduled to undergo unilateral hip replacement surgery at our hospital between June 1, 2023, and June 1, 2024. There are no gender restrictions. The age range of the subjects is 60 - 85 years old, with a weight of 40 - 80 kg. The ASA (American Society of Anesthesiologists) classification is II - III grade.

Exclusion criteria include: history of opioid drug abuse or mental illness, local skin infection at the puncture site, peripheral neuropathy, bradycardia, abnormal coagulation function, and the intake of adrenergic receptor blocking drugs.

A pre-experiment will be carried out initially. Based on the results of these pre-experiment, the number of patients to be finally included in the formal experiment will be determined. The subjects will then be divided into three groups:

Control Group: This group will not receive preemptive analgesia before spinal anesthesia.

Low-concentration PENG Group: Subjects in this group will receive 20 ml of 0.375% ropivacaine.

High-concentration PENG Group: These subjects will be administered 20 ml of 0.5% ropivacaine.

Efficacy Assessment

VAS (Visual Analogue Scale) Scores: Record VAS scores when the patient enters the operating room, during positioning, and after positioning. Also, obtain the anesthesiologist's satisfaction score regarding the patient's position and time of spinal anesthesia operation.

Physiological Parameters: Monitor blood pressure, mean arterial pressure, and heart rate.

Analgesic and Motor Block Parameters: Determine the effective analgesia time and the effective motor block time after surgery. Also, record the incidence of adverse reactions.

Urination - related Metrics: Evaluate the first urination time after surgery. Calculate the incidence of urinary retention after the removal of the urinary catheter, the retention rate of the urinary catheter, and the urinary system infection rate.

Statistical analysis will be performed using SPSS 19.0 software.

Quantitative Data: For quantitative data that follow a normal distribution, they will be presented as mean ± standard deviation. Repeated measurement design data will be analyzed using repeated measures analysis of variance. To compare differences between groups at each time point, the two independent sample t - test will be used. If there is no interaction, only the main effect will be analyzed.

Count Data: Count data will be expressed as cases (%) and group comparisons will be made using the χ2 test. A two - sided P < 0.05 will be considered statistically significant.
